# Prognostic value of CAIX expression in oral squamous cell carcinoma: a systematic review and meta-analysis

**DOI:** 10.1080/14756366.2020.1772250

**Published:** 2020-05-29

**Authors:** Alejandro I. Lorenzo-Pouso, Mercedes Gallas-Torreira, Mario Pérez-Sayáns, Cintia M. Chamorro-Petronacci, Oscar Alvarez-Calderon, Bahi Takkouche, Claudiu T. Supuran, Abel García-García

**Affiliations:** a Oral Medicine, Oral Surgery and Implantology Unit, Faculty of Medicine and Odontology, The Health Research Institute Foundation, University of Santiago de Compostela, Santiago de Compostela, Spain; b Department of Health Sciences, University of Coruña, A Coruña, Spain; c Department of Preventive Medicine, University of Santiago de Compostela, Santiago de Compostela, Spain; d Department of NEUROFARBA, Section of Pharmaceutical and Nutraceutical Sciences, University of Florence, Florence, Italy

**Keywords:** Mouth neoplasm, carbonic anhydrase, meta-analysis, prognosis, hypoxia

## Abstract

Carbonic anhydrase IX (CAIX) is a hypoxia-related protein considered as a predictor for oral squamous cell carcinoma (OSCC) biological behaviour. Nevertheless, this prognostic value is still yet to be validated. We aim to quantify prognostic significance of CAIX overexpression in OSCC by meta-analysis. We performed searches in MEDLINE, EMBASE, SCOPUS, WOS, WHO’S databases, CPCI, and OATD from inception to August 2019. Overall survival (OS), disease-free survival (DFS), locoregional control (LC), and disease-specific survival (DSS) were considered as outcomes of interest. Overall 18 studies were included. CAIX overexpression was associated with worse OS (hazard ratio [HR] = 1.45 95% confidence interval [CI] 1.17–1.80) and DFS (HR = 1.98 95% CI 1.18–3.32). To the contrary, it was neither associated with LC (HR = 1.01 95% CI 0.50–2.02) nor with DSS (HR = 1.35 95% CI 0.78–2.33). Heterogeneity was negligible in all analyses except for DSS. Small studies effect was not significant for OS and DFS. This study shows that immunohistochemical CAIX assessment is a useful OSCC prognostic biomarker.

## 1. Introduction

Hypoxia is one of the most complex conditions that cellular and extracellular matrix confront in order to preserve their homeostasis^1^. Bearing the hallmarks of cancer in mind as described by Hanahan and Weinberg, hypoxic metabolic reprogramming elicits complex mechanisms which take place in the microenvironment of many solid tumours^2^^,^^3^. Two of the key transcription factors that play major roles in this metabolic reprogramming are the hypoxia inducible factors (HIFs), namely HIF-1α and HIF-2α^4^. Carbonic anhydrase IX (CAIX) is a HIF-1α-dependent protein that regulates cellular and extracellular pH homeostasis under hypoxia, playing a pivotal role in carcinogenesis and in the malignant transition of premalignant disorders^5–7^. Several studies have evaluated the prognostic value of CAIX in different types of cancer, including oral squamous cell carcinoma (OSCC)^8^^,^^9^.

OSCC accounts for 95% of all oral malignant neoplasms and its global five-year survival rate ranges between 50 and 60%^10^. The TNM cancer staging system is still considered to be of great prognostic value for this solid tumour^11^. Nonetheless, this system cannot be used to accurately predict the biologic properties of OSSC, nor can it be used to provide guidance for treatment strategies from a molecular biology perspective. Analysing molecular alterations in order to detect specific abnormalities at a transcriptional, translational, and post-translational level has proven to be of particular interest^12^^,^^13^. In this line, immunohistochemistry (IHC) – the use of mono and polyclonal antibodies to determine the tissue distribution of an antigen – has an outstanding oncological impact^14^. Nonetheless, the clinical translation of these IHC-based approaches in terms of decision-making, remains suboptimal for many solid tumours^14^^,^^15^.

There is growing evidence which confirms the key role of CAIX in oral oncogenesis. Numerous publications have explored a possible relationship between CAIX expression and OSCC prognosis, as well as the rate of progression from oral potentially malignant disorders to OSCC^16–18^.

The only meta-analysis carried out on this issue so far did not assess the prognostic value in subgroups^19^. It is known that the prognosis of head and neck cancers is different through tumour locations. It is then of paramount importance to carry out subgroup analyses^20^.

We, therefore, decided to perform a systematic review and meta-analysis on the influence of CAIX expression on the long-term outcomes of patients suffering from OSCC.

## 2. Material and methods

### 2.1. Protocol and eligibility criteria

A systematic literature review was conducted in November 2019 and the protocol used adhered to the PRISMA guidelines^21^. The search question was formulated according to the PECO framework, and it read as follows: What is the prognostic value of tumoural CAIX immunohistochemical expression in patients with OSCC?

### 2.2. Sources

Electronic searches were carried out in MEDLINE *via* PubMed, EMBASE *via* OVID, Web of Science, Scopus, the WHO five regional bibliographic databases (AIM, LILACS, IMEMR, IMSEAR, and WPRIM), and the Conference Proceedings Citation Index databases. For Medline, the following algorithm was used both in the Medical Subject Heading and in the free text words: (“CAIX”) OR (“ca9”) OR (“carbonic anhydrase IX”) OR (“carbonic anhydrase 9”) OR (“carbonic anhydrase-IX”) OR (“carbonic anhydrase-9”) OR (“CA-IX”) OR (“ca-9”) OR (“G250”) AND (“carcinoma, squamous cell” OR “carcinoma” AND “squamous” AND (“cell”) OR “squamous cell carcinoma”) OR (“mouth neoplasm”). The aforementioned syntax was conveniently adapted for each database. All of the databases were searched from inception to August 2019. This process was complemented by a manual search in a series of peer-reviewed journals with related content. Potentially relevant articles that any of the authors were familiar with, as well as reference lists from the retrieved articles, were also comprehensively checked. In these searches, no language restrictions were applied.

### 2.3. Study selection and data extraction process

The study eligibility criteria were applied independently by two trained reviewers (A.I.L.P. and M.P.S.). Any discrepancies were resolved by consensus of all participating authors.

Criteria for eligibility for retrieved studies in the qualitative/quantitative analysis were as follows: i) original research articles published in any language; ii) assessing CAIX expression in biopsies from patients with OSCC using IHC methods; iii) analysing the association between CAIX overexpression with any of the following long-term outcomes: overall survival (OS), disease-free survival (DFS), locoregional control (LC), and disease-specific Survival (DSS). The exclusion criteria were as follows: i) case reports, editorials, or letters; *in vitro* or animal-based studies; ii) insufficient statistical data to estimate predefined outcomes; iii) studies evaluating CAIX protein-related genes or miRNAs; iv) studies with duplicated cohorts.

In the first round, the title and abstract of the retrieved articles and studies which met the inclusion criteria were read and any texts which presented insufficient data in order for a clear decision to be made were assessed following a full-text protocol. Subsequently all of the studies which were considered eligible were fully examined in a second round and the final decision as to whether or not they were to be included was made. This form included the following items: first author, year of publication, country and continent where the study was conducted, sample size, recruitment period, tumour subsite, treatment modality, follow-up period, cut-off value for CAIX IHC positivity, immunostaining pattern (nuclear/cytoplasmic), hazard ratios (HRs) for long-term outcomes, and adjustment variables.

### 2.4. Quality assessment, data synthesis, and analysis

Quality was independently assessed by two authors (O.A.C. and C.M.C.P.) by means of a variation of the criteria formulated in the Reporting Recommendations for Tumour Marker Prognostic Studies (REMARK) guidelines for prognostic studies and the Standards for Reporting of Diagnostic Accuracy (STARD) developed by Troiano et al^22^. This variation included six dimensions which evaluated:

1. Samples: i) Cohort (retrospective or prospective) study with a well-defined study population; ii) Medical treatment applied to the patients was explained. Authors have explained if all patients have received the same treatment or not.
2. Clinical data of the cohort: The basic clinical data such as age, gender, clinical stage, and histopathological grade was provided.
3. IHC: Well-described staining protocol or referred to original paper.
4. Prognosis: The analysed survival endpoints were well defined (e.g. OS and DFS).
5. Statistics: i) Cut-off point, which is used to divide the cases into risk groups was well described; ii) Estimated effect describing the relationship between the evaluated biomarker and the outcome was provided; (iii) Adequate statistical analysis (e.g. Cox regression modelling) was performed to adjust the estimation of the effect of the biomarker for known prognostic factors.
6. Classical prognostic factor: The prognostic value of other classical prognostic factors and its relationship with the studied factor was reported.

Each parameter could be identified by one of three attributes (i.e. adequate [A], inadequate [I], or non-evaluable [N/A]. Each item scored as adequate adds one point to overall quality assessment for each study. A score sheet was prepared for each included study and quality scoring was independently undertaken by aforementioned author. In the event of disagreement, the scores were discussed until a consensus was reached. Studies were categorised as high quality when the overall score was >4.

The differences in the levels of CAIX staining were categorised as high and low, according to the cut-off value which was chosen by the authors of the studies. HRs and 95% confidence intervals (CIs) were used as the measure of association in order to estimate the impact of CAIX expression on the aforementioned long-term outcomes (OS, DFS, LC, and DSS). Multivariate or univariate HRs values were used but, when available, the formers were chosen. When data on the HRs could not be directly traced, these were calculated using the approximation methods described by Parmar et al.^23^ and Tierney et al.^24^. Lastly, when access was provided to full databases, the HRs were directly extracted.

Pooled analyses were obtained using both fixed-effect models (i.e. Mantel–Haenszel method) and random-effect models (i.e. DerSimonian and Laird method), but when substantial heterogeneity was detected, we based our assessment only on random-effects models. A subgroup analysis on the basis of several variables was planned (i.e. quality score, ethnic variations, tumour subsite, CAIX antibody, cut-off point, and type of covariate adjustment). The heterogeneity was assessed using the proportion of the total variance due to the variance between studies (R_i_)^25^. Large values (>0.75) indicate a large amount of heterogeneity, values between 0.4 and 0.75 suggest a moderate amount, whereas small values (<0.4) indicate a low amount of heterogeneity. Funnel plots were used to visually assess publication bias and the Egger’s test was used in order to conduct a more formal analysis. Stata version 14.1 (Stata Corp, College Station, TX, US) and HEpiMA version 2.1.3 (Corunna, Galicia, Spain) were employed^26^.

## 3. Results

### 3.1. Characteristics of the included studies

**Figure 1.**
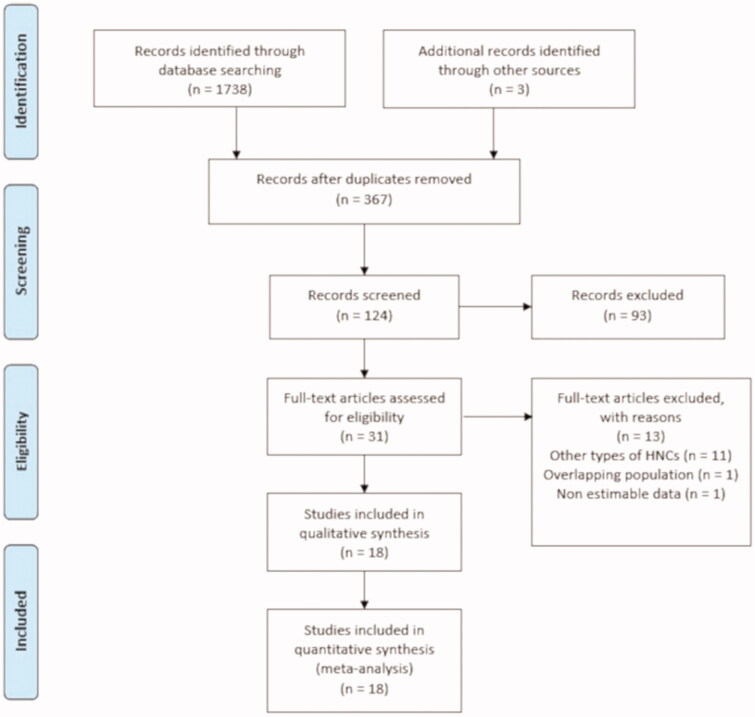
Flowchart for inclusion of the studies according to the PRISMA guidelines.

Out of the 1741 publications which were initially retrieved, 18 studies met our inclusion criteria and were included in the meta-analysis, as depicted in Figure 1. The studies reported on a total of 1616 OSCC-affected patients^27–44^.

**Figure 2.**
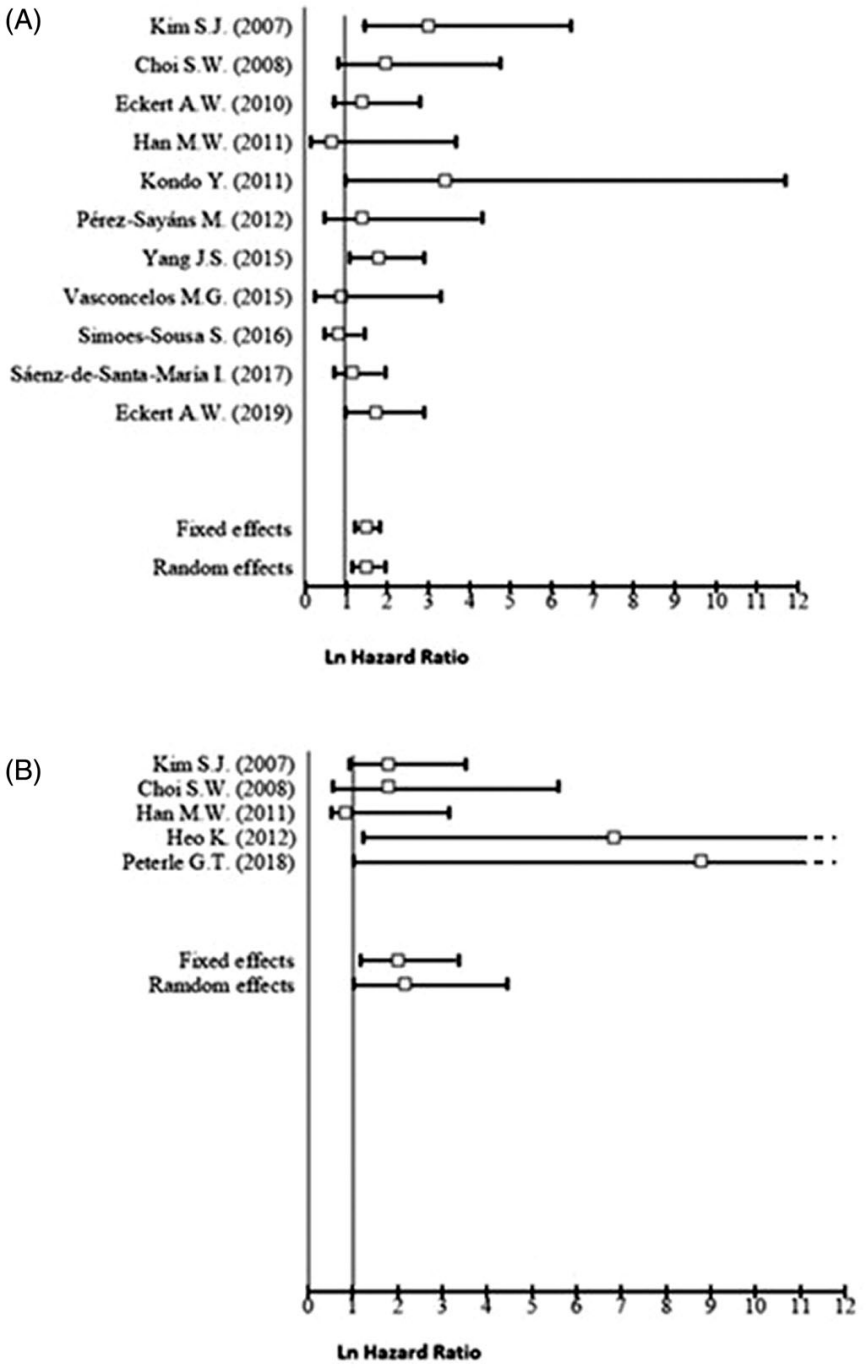
Forest plot for the association of higher CAIX expression with overall survival (A), disease‐free survival (B).

The data was collected in a period spanning from 1987 to 2015^27–44^ while the year of publication ranged from 2008 to 2019. The anatomic location of the tumours was mainly divided into exclusively tongue^27^^,^^28^^,^^30^^,^^31^^,^^37^^,^^39^^,^^42^, or mixed subsite tumours ^29^^,^^32–36^^,^^38^^,^^40^^,^^41^. The population sample ranged from 21 in Roh et al. study^30^ to 271 in Yang et al. study^38^. The studies were carried out in Asia, Europe, and America. Half of the studies were carried out in Asia (specifically in China^38^, Japan^27^^,^^33^, and South Korea^28–30^^,^^32^^,^^35^^,^^37^) and the other half in Brazil^39^^,^^43^, Canada^34^, Germany^31^^,^^44^, Portugal^40^, Spain^36^^,^^40^^,^^42^, and the US^41^. The retrieved data are summarised in Table 1. The individual HRs for each selected long-term outcome with its respective adjustment is reflected in Table 2. The authors had full access to three full databases^36^^,^^40^^,^^42^. An article-based doctoral dissertation retrieved *via* LILACS needed to be fully assessed for its HR estimation^39^.

**Table 1.** Characteristics of the included studies.

Study	Year	Country	Sample size	Tumour subsite	Recruitment period	Treatment	Follow-up (months)	CAIX antibody	IHC pattern	Cut-off point (%)	CAIX (+)
Sakata K et al. [27]	2008	Japan	68	Tongue	1987–2004	Rx	11–146 (mean 56)	Rabbit polyclonal antibody to CAIX (Novus Biologicals)	Membrane	10	32.35% (*n* = 22)
Kim SJ et al. [28]	2007	South Korea	60	Tongue	1997–2004	Sx, and Rx	4.1–117.13 (mean 29.51)	Anti-CAIX mouse monoclonal antibody clone M75	Cytoplasmic membrane	10	63.33% (*n* = 38)
Choi SW et al. [29]	2008	South Korea	117	Buccal mucosa, gingiva, tongue, retromolar trigone, palate, and lip.	1996–2000	Sx, and Rx	2–120 (mean 39.5)	Anti-CAIX mouse monoclonal antibody clone M75	Cytoplasmic membrane	5	58.12% (*n* = 68)
Roh JL et al. [30]	2009	South Korea	21	Tongue	1997–2005	Sx, and Rx	37–123 (mean 60)	Monoclonal antibody CAIX (AF 2188)	Cytoplasmic membrane	10	60.47% (*n* = 26)
Eckert AW et al. [31]	2010	Germany	80	Hard and soft palate, buccal mucosa, tongue, floor of the mouth, mandibular angle, and lower alveolar mucosa	1994–1999	Sx	60	Monoclonal antibody CAIX (HI-20, SC25599)	Cytoplasmic membrane	10	42.5% (*n* = 34)
Han MW et al. [32]	2011	South Korea	33	Tongue	2001–2006	Sx, and Rx	40 (range 9–113)	Monoclonal antibody CAIX (AF 2188)	Cytoplasmic membrane	10	63.6% (*n* = 21)
Kondo Y et al. [33]	2011	Japan	107	Tongue, gingiva, floor of mouth, lips, and buccal mucosa	1992–2009	Sx	60	Rabbit polyclonal to CAIX (Abcam 15086)	Membrane	10	91.58% (*n* = 98)
Brockton NT et al. [34]	2012	Canada	61	Unspecified oral cancer	1998–2005	Sx and Rx	60	Rabbit polyclonal to CAIX (Abcam 15086)	N/A	*	26.23% (*n* = 16)
Heo K. [35]	2012	South Korea	62	Tongue and others	2003–2006	N/A	52.2 (range 5.75–86.9)	Anti-CAIX mouse monoclonal antibody clone M75	Cytoplasmic membrane	10	69.35% (*n* = 41)
Pérez-Sayáns M et al. [36]	2012	Spain	50	Buccal mucosa, soft palate, gums, retromolar trigone, tongue, and floor of the mouth	2006–2010	Rx, Qx, and Sx	28.7–37.9	Anti-CAIX mouse monoclonal antibody clone M75	Cytoplasmic membrane	10	82.00% (*n* = 41)
Hwa JS et al. [37]	2015	South Korea	24	Tongue	1998–2009	Rx, Qx, and Sx	11–116 (mean 56)	Rabbit polyclonal antibody to CAIX (Novus Biologicals)	Membrane	10	20.83% (*n* = 5)
Yang JS et al. [38]	2015	Taiwan	271	Unspecified oral cancer	2000–2006	N/A	150 months	Anti-CAIX antibody (Santa Cruz Biotechnology)	N/A	N/A	41.69% (*n* = 113)
Vasconcelos MG et al. [39]	2015	Brazil	57	Tongue	1995–2007	N/A	N/A	Anti-CAIX antibody (Santa Cruz Biotechnology)	Cytoplasmic membrane	10	66.7% (*n* = 38)
Simoes-Sousa S et al. [40]	2016	Brazil and Spain	135	Tongue, floor of mouth, buccal mucosa, gingiva, and retromolar trigone	NA	Rx, Qx, and Sx	105 months	Antibody CAIX (Abcam 15086)	Cytoplasmic membrane	5	57.78% (*n* = 78)
Brockton NT et al. [41]	2017	USA	168	Tongue, floor of mouth, buccal mucosa, and gingiva	2003–2012	Rx, Qx, and Sx	33 (range 0.2–111.0)	Rabbit polyclonal CAIX (Abcam 15086)	N/A	*	25.00% (*n* = 42)
Sáenz-de-Santa-María I et al. [42]	2017	Spain	108	Tongue	N/A	N/A	N/A	Antibody Carbonic CAIX (Abcam 15086)	Cytoplasmic membrane	23	48.15% (*n* = 52)
Peterle GT et al. [43]	2018	Brazil	52	Unspecified oral cancer	2002–2008	Sx and Rx	24–60 months	Antibody CAIX (Abcam 108351)	Cytoplasmic membrane	25	50.00% (*n* = 26)
Eckert AW et al. [44]	2019	Germany	158	Unspecified oral cancer	1997–2015	Sx	105 months	Anti-CAIX mouse monoclonal antibody clone M75	N/A	51	15.19% (*n* = 24)

N/A: not available; Qx: chemotherapy; Rx: radiotherapy; Sx: surgery

*Raw AQUA score distributions for CAIX were evaluated: high CAIX expression was defined as an AQUA score within the upper quartile. Low CAIX expression was defined as an AQUA score within the lower three quartiles.

**Table 2.** Synthesis of data extracted from the included studies related to outcomes pooled in the meta‐analysis.

Study	OS (HR 95% CI)	DFS (HR 95% CI)	LC (HR 95% CI)	DSS (HR 95% CI)	Adjustment
Sakata K et al. [27]	NR	NR	0.91 (0.32–2.61)	NR	Multivariate adjusted for T stage and microvessel density.
Kim SJ et al. [28]	2.99 (1.39–6.45)	1.76 (0.89–3.51)	NR	NR	None
Choi SW et al. [29]	1.91 (0.77–4.71)	1.77 (0.56–5.56)	NR	NR	None
Roh JL et al. [30]	NR	NR	1.09 (0.43–2.76)	0.71 (0.23–2.22)	None
Eckert et al. [31]	1.34 (0.65–2.76)	NR	NR	NR	Multivariate adjusted for tumour size and tumour grade.
Han MW et al. [32]	0.65 (0.12–3.67)	0.80 (0.50–3.15)	NR	NR	Multivariate adjusted for tumour size and tumour grade.
Kondo Y et al. [33]	3.36 (0.97–11.70)	NR	NR	NR	None
Brockton NT et al. [34]	NR	NR	NR	2.96 (1.01–8.66)	Multivariate adjusted for tumour stage and nodal involvement.
Heo K et al. [35]	NR	6.82 (1.22–37.94)	NR	NR	None
Pérez-Sayáns M et al. [36]	1.36 (0.43–4.26)	NR	NR	2.04 (0.76–5.49)	None
Hwa JS et al. [37]	NR	NR	NR	0.29 (0.05–1.77)	None
Yang JS et al. [38]	1.76 (1.07–2.87)	NR	NR	NR	None
Vasconcelos MG et al. [39]	0.86 (0.23–3.26)	NR	NR	NR	None
Simoes-Sousa S et al. [40]	0.76 (0.44–1.38)	NR	NR	NR	None
Brockton NT et al. [41]	NR	NR	NR	1.0 (1.00–1.01)	Multivariate adjusted for tumour stage and nodal involvement.
Sáenz-de-Santa-María I et al. [42]	1.14 (0.69–1.89)	NR	NR	NR	Multivariate adjusted for tumour stage and nodal involvement.
Peterle GT et al. [43]	NR	8.75 (0.99–77.19)	NR	2.84 (1.02–7.87)	Multivariate adjusted for tumour size and the use of Rx.
Eckert AW et al. [44]	1.70 (0.97–2.85)	NR	NR	NR	Multivariate adjusted for tumour stage, lymph node status, and tumour grade.

NR: no report.

### 3.2. Quality assessment and pooled analysis of the included studies

The quality assessment which was performed in accordance with the REMARK guidelines is summarised in Table 3. According to these criteria ten studies showed a good quality, although eight were considered at high risk of bias. Table 4 lists the pooled effect estimates for all 18 studies, for each of the selected long-term outcomes, as well as the analysis of its subgroups. A fixed-effects model was used to evaluate the pooled HR with 95% CI for the outcomes of OS, DFS, and LC, given that they displayed low heterogeneity. A random-effects model was used for DSS due to its high heterogeneity. A higher CAIX expression is associated with a statistically significant worse OS (HR = 1.45, 95% CI 1.17–1.80), and in the case of DFS, the pooled analysis reflected almost a two-fold increase in the hazard for this outcome (HR = 1.98, 95% CI 1.18–3.32). However, higher CAIX expression is apparently not related to LC (HR = 1.01, 95% CI 0.50–2.02), nor to DSS (HR = 1.35, 95% CI 0.78–2.33).

**Table 3.** Quality score according to the REMARK guidelines.

Study	Year	Samples	Clinical data of the cohort	Immunohisto chemistry	Prognosis	Statistics	Classical prognostic factors	Overall risk of bias
Sakata K et al. [27]	2008	A	I	A	I	I	I	2
Kim SJ et al. [28]	2007	A	A	A	A	A	A	6
Choi SW et al. [29]	2008	A	A	A	I	A	A	5
Roh JL et al. [30]	2009	A	A	A	A	A	A	6
Eckert AW et al. [31]	2010	A	A	A	N/A	I	I	3
Han MW et al. [32]	2011	A	I	A	I	A	A	4
Kondo Y et al. [33]	2011	A	A	A	A	A	A	6
Brockton NT et al. [34]	2012	A	I	A	A	A	A	5
Heo K et al. [35]	2012	I	A	A	I	A	A	4
Pérez-Sayáns M et al. [36]	2012	A	A	A	I	A	A	5
Hwa JS et al. [37]	2015	I	A	A	I	I	I	2
Yang JS et al. [38]	2015	I	A	A	I	A	A	4
Vasconcelos MG et al. [39]	2015	A	I	A	I	I	A	3
Simoes-Sousa S et al. [40]	2016	I	A	A	A	A	A	5
Brockton NT et al. [41]	2017	A	A	A	A	A	A	6
Sáenz-de-Santa-María I et al. [42]	2017	A	I	A	A	A	I	4
Peterle GT et al. [43]	2018	A	A	A	I	A	A	5
Eckert AW et al. [44]	2019	A	A	A	A	A	A	6

Items were assessed as A: Adequate; I: Inadequate; N/A: no description.

**Table 4.** Pooled hazard ratios and 95% confidence intervals.

	Number of studies	Pooled HR (95% CI), fixed effects	Pooled HR (95% CI), random effects	R_i_^*^	Q test *p* Value
Overall survival					
Overall	11	1.45 (1.17–1.80)	1.46 (1.12–1.89)	0.26	.21
High quality	6	1.48 (1.18–1.86)	1.51 (1.11–2.06)	0.38	.13
Low quality	5	1.21 (0.64–2.29)	1.21 (0.64–2.29)	0.00	.57
Full adjustment	4	1.48 (1.18–1.86)	1.51 (1.11–2.06)	0.38	.13
Asian	5	2.01 (1.42–2.86)	2.01 ( 1.42–2.86)	0.00	.45
Non-Asian	6	1.19 (0.91–1.57)	1.19 (0.91–1.57)	0.00	.45
Tongue	4	1.18 (0.84–1.66)	1.22 (0.66–2.27)	0.66	.04
Mixed subsites	7	1.48 (1.14–1.91)	1.48 (1.10–2.00)	0.22	.29
Use of M75 antibody	3	1.93 (1.29–2.88)	1.93 (1.29–2.88)	0.00	.39
Use of other antibodies	8	1.29 (1.00–1.67)	1.29 (0.96–1.75)	0.21	.27
Use of 10% cut-off point	6	1.72 (1.14–2.59)	1.69 (1.06–2.69)	0.17	.31
Use of other cut-off points	5	1.36 (1.06–1.75)	1.36 (0.98–1.88)	0.39	.17
Disease-free survival					
Overall	5	1.98 (1.18–3.32)	2.12 (1.02–4.43)	0.40	.19
Asian	4	1.81 (1.06–3.09)	1.82 (0.90–3.69)	0.34	.25
Tongue	2	1.51 (0.79–2.88)	1.27 (0.43–3.74)	0.57	.21
Mixed subsites	3	3.24 (1.35–7.76)	3.58 (1.26–10.12)	0.26	.39
Use of 10% cut-off point	3	1.82 (0.99–3.33)	1.85 (0.59–5.77)	0.65	.12
Use of other cut-off points	2	2.50 (0.91–6.89)	2.98 (0.69–12.91)	0.48	.20
Locoregional control					
Overall	2	1.01 (0.50–2.02)	1.01 (0.50–2.02)	0.00	.80
Disease specific survival					
Overall	6	1.00 (0.99–1.01)	1.35 (0.78–2.33)	1.00	.03
Asian	2	0.55 (0.21–1.44)	0.55 (0.21–1.44)	0.00	.41
Non-Asian	4	1.00 (0.99–1.01)	1.79 ( 0.90–3.54)	1.00	.01
Tongue	2	0.55 (0.21–1.44)	0.55 (0.21–1.44)	0.00	.41
Mixed subsites	4	1.00 (0.99–1.01)	1.79 (0.90–3.54)	1.00	.02

*R_i_ stands for the proportion of the total variance due to between studies variance.

**Figure 3.**
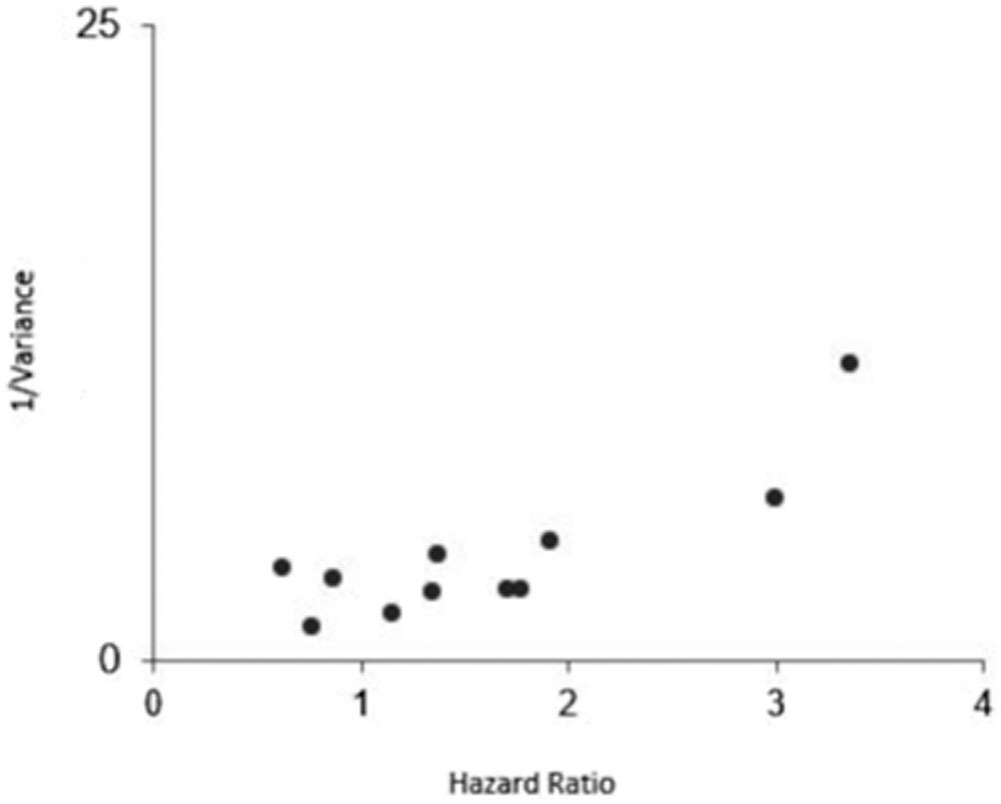
Funnel plot of publication bias for higher CAIX expression and overall survival.

The funnel plot for OS (Figure 3) indicates a minimal skewness to the right that was not confirmed by the Egger’s test (P_Egger_ = 0.59). In the case of DFS, this statistical test also indicates the absence of publication bias (P_Egger_ = 0.29).

In the OS subgroup analysis, the statistically significant association was not preserved in the non-Asian (HR = 1.19, 95% CI 0.91–1.57), low quality (HR = 1.21, 95% CI 0.64–2.29), and tongue carcinoma subgroups (HR = 1.18, 95% CI 0.84–1.66). The analysis showed that the pooled HR was twice higher for the Asian group (HR = 2.01, 95% CI 1.42–2.86) when compared to the Non-Asian group. Furthermore, the use of M75 antibody and the choice of a 10% cut-off point were related with increased HR, [HR = 1.93, 95% CI 1.29–2.88], and HR =1.72, 95% CI 1.14–2.59), respectively. In the case of DFS, its statistical significance was preserved in the Asian group (HR = 1.81, 95% CI 1.06–3.09), mixed subsites (HR = 3.24, 95% CI 1.35–7.76), and use of other cut-off points than 10% for IHC subgroups (HR = 2.50 95% CI 0.91–6.89) (Figure 2).

## 4. Discussion

Globally, this systematic review and meta-analysis show that CAIX overexpression is correlated with worse OS and DFS in OSCC patients, indicating that positivity for this test implies that the overall risk of dying increases by about 50%. The relation between CAIX expression and OS and DFS appears to confirm the prognostic value that is attributed to this marker on the basis of its relation with the HIF pathway^9^^,^^45^. CAIX contributes to the tumour microenvironment by maintaining extracellular acidic pH and helping cancer cells grow and metastasise in several other solid tumours^5^^,^^6^.

Our study showed a lower prognostic value for OS and DFS than that shown in Peridis et al.’s study^19^. Our study may be more reliable due to the accumulation of studies with high-quality scores and lower heterogeneity across studies. In addition, to our knowledge, our study is the first meta-analysis to measure this prognostic value exclusively in OSCC.

The relative consistency of the results across subgroups reinforces the plausibility of the findings. First, publication bias is a highly unlikely explanation for the present results given the findings of the asymmetry tests of the funnel plot for both OS and DFS (Figure 3). It is worth mentioning that we observed that a relevant part of the studies included in our meta-analysis did not provide HRs estimates adjusted for relevant cofounders, although subgroup analysis demonstrated that even after the use of fully adjusted models for multiple established OSCC risk factors the OS remained with the same magnitude. Also, the hypoxic nature of these tumours is highly influenced by the crosstalk with other molecular pathways such cell cycle or angiogenesis, and in our review several reports took these factors into account^9^.

Second, the heterogeneity of the studies included in the present meta-analysis was generally small especially in relation to LC, DFS, and OS (Table 4). On the contrary, there was high heterogeneity in DSS. We relate this finding to the fact that survival parameters were undefined in some studies, largely due to the lack of international consensus on the definitions of long-term outcomes. In the OS subgroup analysis, CAIX overexpression also had a particularly negative impact on the OS of Asian patients. These differences could be linked to genetic and lifestyle variabilities^10^. This study also identified that variations at the level of staining protocol also resulted in significant variations in survival endpoints. In the case of the DFS subgroup analysis, a stronger association in the OSCC mixed subsites subgroup, when compared exclusively with the tongue carcinoma subgroup, was observed. Prognostic OSCC studies have classically described tongue carcinomas as those tumours which carry the worst prognosis due to the lymphatic richness and the more pronounced diagnostic delay^9–12^. We relate this finding to the possible existence of residual confounding given that some studies pooled data regarding unspecified oral cancer subsites.

This study dealt with the IHC-based CAIX expression in OSCC tissue as a prognostic but not a predictive biomarker. Nonetheless, recent studies have shed light on its promising value as a predictor of the malignant transformation of some orally potentially malignant disorders, such as oral leukoplakia^16^. CAIX as a target is of particular interest in oncology as there are a number of CAIX inhibitors available^7^. The interference of the HIF pathway with these inhibitors in OSCC has been poorly explored, nonetheless, according to the recent studies carried out by our group, several CAIX inhibitors which were synthesised on phenolic bis mannich bases and sulphonamides showed promising *in vitro* cytotoxicities in several OSCC cell lines^46–49^.

The results from this systematic review and meta-analysis are supported by strong evidence. However, when interpreting the main results from this meta-analysis, some of the limitations of the individual studies cannot be ignored. Some authors recorded ambiguity in the distinction between OS and DSS. In addition, some authors did not directly report HR values in the survival analysis and this had to be approximated. In addition, adjustment for multiple established OSCC risk factors varied widely in the included studies. Despite this, we believe that these results are reliable and more widely applicable. However, further immunohistochemical reports are needed in order to validate this biomarker.

## 5. Conclusions

In view of the results obtained, we believe that IHC assessment of CAIX expression may be useful as a prognostic biomarker for OSCC, especially in the case of OS and DFS. These results open up the possibility of using this hypoxia-related protein in the prognosis of OSCC, and in its prevention and early control. Future studies with larger sample sizes and well-designed inclusion criteria are warranted in order to assess the role of CAIX IHC-based expression in determining the prognosis of OSCC.
